# Consensus Terminology for Point of Care Ultrasound Studies with Incomplete Documentation and Workflow Elements

**DOI:** 10.24908/pocus.v7i1.15017

**Published:** 2022-04-21

**Authors:** Jason T Nomura, Matthew Flannigan, Rachel B Liu, Daniel L Theodoro

**Affiliations:** 1 Dept of Emergency Medicine, ChristianaCare Newark, DE USA; 2 Dept of Emergency Medicine, Spectrum Health/Michigan State University USA; 3 Dept of Emergency Medicine, Yale School of Medicine USA; 4 Dept of Emergency Medicine, Washing University School of Medicine USA

**Keywords:** ultrasonography, terminology, definition, workflow, consensus, research

## To the Editor:

Point of Care Ultrasound (POCUS) use by emergency physicians has grown in both breadth and depth of clinical use 1, 2, 3. POCUS workflow is different from a traditional imaging-based specialist workflow because a single clinician orders, obtains images, interprets, and reports the exam results. Traditionally, multiple individuals participate in the workflow: the clinician places the order; an ultrasound technologist reconciles order and identifiers and acquires images; lastly an imaging specialist interprets the exam and creates a report. These contrasting approaches has led to unique challenges in streamlining POCUS workflows and identifying disruptions, errors and potential corrections given the differences from radiology focused workflow and resources.

There have been limitations in institutional support and resources to create infrastructure to support POCUS exams and documentation workflows 4, 5. This has also not been a primary focus of research and the literature has a variety of terminology to describe workflow and documentation errors 6. Enhancing the availability of POCUS images and the dissemination of reporting is an essential component for continuity of patient care and will continue to gain importance as POCUS continues to grow in Emergency Medicine and other specialties outside of traditional imaging specialties.

The objective of this study was to develop a standard terminology for workflow related errors in POCUS documentation. Standardizing terminology is important because it serves as the basis for future research to address and prevent workflow disruptions which can affect data transparency impacting patient safety and quality of care. Standardized terminology in technical standards such as the Systematized Nomenclature of Medicine – Clinical Terms (SNOMED-CT), Integrating the Healthcare Enterprise (IHE), and Health Level Seven (HL7) projects is becoming more pervasive with increased use of technology. Standardized terminology for research topic areas is also increasing to enhance the reproducibility and implementation of research findings 7. This protocol was approved by the ChristianaCare Institutional Review Board. This study was initially part of a Society of Clinical Ultrasound Fellowships (SCUF) workshop on POCUS workflow with respondents solicited from SCUF through the membership listserv. 

Respondents were presented with the premise that a completed POCUS exam needs to have the following elements: images; patient identifiers; an identified operator, and a report interpretation. We did not specify the number of identifiers or the elements needed in an interpretation report as this can vary by institution, software, and type of study. 

An electronically distributed modified Delphi process was used to define standard terminology for POCUS studies with missing elements due to workflow errors. The first phase presented current terminology utilized by the authors and colleagues for selection and requested any additional terminology used by participants. As an example question, for studies with images and documentation, but missing patient identifiers respondents were initially given the options of “incomplete study missing patient identifiers”, “partial study missing patient identifiers”, “phantom study”, “ghost study”, or “unlabeled study” or to submit other potential terms. A particular error, with many names, that can occur during POCUS utilization is the performance of an examination without captured images or documentation making it a difficult error to identify and quantify as the exam does not exist in either the medical record or image archiving software. 

During phase 2 the respondents chose to endorse the most common terms from phase 1 or selected from other potential terminology in a First-Pass-the-Post or an Instant-Runoff voting methodology, depending on the number of options to speed agreement. Phase 3 presented the consensus terminology to the volunteer group and then the broader SCUF membership for acceptability. 

Volunteers were solicited from the 122 ultrasound fellowship programs included in the SCUF listserv. The listerv consists of the 122 program directors and any faculty that become SCUF members, the total membership is not publicly available. Phase 1 had 53 respondents who agreed to participate in the modified Delphi process. The group included 28 Ultrasound Directors, 31 Fellowship Directors, 24 Core US Faculty, 9 Associate or Assistant Directors; positions are not mutually exclusive, representing 43 unique programs or 35% of fellowships at the time of the survey. The 2^nd^ and third phases were only distributed to the volunteer group of 53 faculty from phase 1. Thirty of the original 53 (57%) volunteers replied to the 2^nd^ phase. All but three (90%) chose to use the term “incomplete or partial study” with a qualifier. Of those 27 individuals 25 (93%) chose “incomplete” as the preferred term. Those that did not choose “incomplete” as their first choice ranked it as their 2^nd^. For studies that have no retained images or documentation, and by electronic means does not exist, the term “phantom scan” was the primary choice by 21 respondents (70%) over “ghost scan”. The terminology was then presented to the volunteer group for review, with 37 responses (70% of volunteer group) unanimously agreeing that the terminology presented in Table 1 was acceptable as consensus terminology. This was then presented to the SCUF membership via listserv with an additional 70 respondents (37 Ultrasound Directors, 41 Fellowship Directors, 15 Associate or Assistant Directors and 25 Core Faculty, categories not mutually exclusive), for a total of 107 unique respondents who unanimously voted that the terminology was acceptable consensus. Respondents represented 63 separate programs or 52% of fellowships at the time of the survey. The final agreed upon terms are not currently delineated in published literature or technical standards. Terminology in published research has some similarities as the study population may have included those authors and they were able to suggest terminology, however the consensus terms have not been previously published. 

**Table 1:** Consensus Terminology for Point of Care Ultrasound Examinations with Missing Documentation and Workflow Elements.

**Term**	**Definition**
Incomplete study, missing operator	Study does not contain information on who performed the study
Incomplete study, missing documentation	Study does not have a complete interpretation report
Incomplete study, missing images	Study does not have complete images
Incomplete study, missing patient identifiers	Study does not contain patient identifiers
Phantom scan	A study that has not been documented in the medical record or have archived images; when audited it does not exist.

Our study presents an agreed upon list of consensus terms to identify POCUS studies that are missing key elements for documentation and archiving as an imaging study. The use of standardized terminology for workflow errors can be utilized for research in point of care ultrasound workflow to find solutions to prevent and correct errors. Use of standard terminology will improve communication to assist in the implementation and interoperability of workflows between institutions, programs and specialties. Consistent terminology can also facilitate the reproducibility, implementation, and expansion of point of care ultrasound workflow research and operational practices across different POCUS programs and specialties. We suggest that these terms be utilized when performing point of care ultrasound workflow research to facilitate understanding of problems, potential solutions and application of interventions.

## Disclosures

JTN has received funding personally from Philips for consulting. RBL has received funding personally from Philips for consulting. MF has no disclosures. DLT has received funding from the Emergency Medicine Foundation for ultrasound research.
